# Unraveling the Strength and Nature of Se∙∙∙O Chalcogen Bonds: A Comparative Study of SeF_2_ and SeF_4_ Interactions with Oxygen-Bearing Lewis Bases

**DOI:** 10.3390/molecules29235739

**Published:** 2024-12-05

**Authors:** Renhua Chen, Fengying Lei, Deze Jin, Ke Peng, Qingyu Liu, Yeshuang Zhong, Liang Hong, Xiaolong Li, Zhu Zeng, Tao Lu

**Affiliations:** 1 School of Basic Medical Sciences/School of Biology and Engineering, Guizhou Medical University, Guiyang 550025, China; chenrenhua5288@163.com (R.C.); leifengying@gmc.edu.cn (F.L.); 18586805183@163.com (D.J.); pengke7463@163.com (K.P.); l.qingyu@foxmail.com (Q.L.); zhongyeshuang@foxmail.com (Y.Z.); hongliang0702@126.com (L.H.); 2 Department of Chemistry, Shanghai Key Laboratory of Molecular Catalysis and Innovative Materials, Fudan University, Shanghai 200433, China; xiaolong_li@fudan.edu.cn

**Keywords:** quantum chemical computations, selenium-centered chalcogen bonds, σ-hole interactions, selenium-containing compounds, binary complexes

## Abstract

Chalcogen bonds (ChBs) involving selenium have attracted substantial scholarly interest in past years owing to their fundamental roles in various chemical and biological fields. However, the effect of the valency state of the electron-deficient selenium atom on the characteristics of such ChBs remains unexplored. Herein, we comparatively studied the σ-hole-type Se∙∙∙O ChBs between SeF_2_/SeF_4_ and a series of oxygen-bearing Lewis bases, including water, methanol, dimethyl ether, ethylene oxide, formaldehyde, acetaldehyde, acetone, and formic acid, using ab initio computations. The interaction energies of these chalcogen-bonded heterodimers vary from −5.25 to −11.16 kcal/mol. SeF_2_ participates in a shorter and stronger ChB than SeF_4_ for all the examined heterodimers. Such Se∙∙∙O ChBs are closed-shell interactions, exhibiting some covalent character for all the examined heterodimers, except for SeF_4_∙∙∙water. Most of these chalcogen-bonded heterodimers are predominantly stabilized through orbital interactions between the lone pair of the O atom in Lewis bases and the σ*(Se–F) antibonding orbitals of Lewis acids. The back-transfer of charge from the lone pair of selenium into the σ* or π* antibonding orbitals of Lewis bases is also observed for all systems. Energy decomposition analysis reveals that the electrostatic component significantly stabilizes the targeted heterodimers, while the induction and dispersion contributions cannot be ignored.

## 1. Introduction

σ-hole interactions [1,2,3], which involve elements from groups IV–VII serving as electrophilic sites, have aroused considerable attention from experimental and theoretical scientists in the past two decades owing to they play a pivotal role in a variety of chemical and biological processes [4,5,6,7,8,9]. Depending on the group of the elements associated with the σ-holes [10,11], which are the positive electrostatic potential regions on the extension of a covalent bond, these σ-hole interactions are called tetrel bonds (TtBs, group IV) [12], pnictogen bonds (PnBs, group V) [13], chalcogen bonds (ChBs, group VI) [14], and halogen bonds (XBs, group VII) [15,16], respectively. Among these σ-hole interactions, experimental and theoretical studies of the XBs have been widely documented in the literature up to now [2,16,17,18,19]. In recent years, researchers have increasingly shifted their attention to ChBs, which represent an attractive interaction between an electron-deficient region associated with a covalently attached group VI atom (O, S, Se, Te) and a Lewis base (e.g., a lone pair donor, a π-electron system, or an anion) acting as an electron donor. This is due to the substantial role that they play in diverse fields such as supramolecular chemistry [20], materials chemistry [21], catalysis [22], organosynthesis [23], crystal engineering [24], anion recognition [4,25], and biological systems [26,27,28]. It was shown that chalcogen atoms can interact with Lewis bases as electrophilic sites (ChB donors) and with Lewis acids as nucleophilic sites (ChB acceptors) in the formation of the ChBs [29]. Compared to hydrogen bonds (HBs) [30], ChBs are more hydrophobic and have higher directionality. Studies have revealed that ChBs are comparable in strength to HBs and XBs. Furthermore, it has also been demonstrated that the combination of the electrostatic, dispersion, charge transfer, and orbital delocalization components is primarily responsible for the physical nature of ChBs [31,32,33].

Currently, the majority of experimental and theoretical studies have centered on ChBs involving the sulfur atom as an electrophilic site, such as S∙∙∙N [34,35], S∙∙∙O [36,37], S∙∙∙F [38,39], S∙∙∙S [40,41], and S∙∙∙π [42,43,44] chalcogen-bonding interactions. However, selenium-centered ChBs should be given special attention because they also show many promising applications in the aforementioned chemical and biological realms [22,23,24,27,45]. Furthermore, statistical analyses of crystal structures from the Protein Data Bank (PDB) and the Cambridge Structural Database (CSD) have revealed the existence of numerous intramolecular and intermolecular selenium-centered ChBs, particularly the Se∙∙∙O ChBs, in organoselenium compounds, proteins, and protein–ligand complexes [27,28,45,46,47,48], thereby further demonstrating their significance. For instance, Metrangolo and colleagues have suggested that Se∙∙∙O ChBs are ubiquitous in a multitude of selenomethionine-containing proteins by conducting a statistical analysis of crystal structures from the PDB, underscoring their importance in manipulating the structures and functions of proteins and peptides [27]. It is noteworthy that these nucleophilic oxygen atoms located in the protein backbone or the side chains of amino acids exhibit either sp^2^ or sp^3^ hybridization.

Nowadays, most studies [24,27,28,32,45,47,48,49,50,51,52,53] on selenium-centered ChBs have focused on the divalent bonding situation of Se such as SeF_2_ and selenoethers. However, analogous to its sister element S, the Se atom can frequently exhibit tetravalent bonding in selenium-containing compounds such as SeO_2_ and SeF_4_. These compounds as ChB donors are also capable of participating in ChBs by interacting with various Lewis bases. For example, Ehm and coauthors [54] have revealed that the stability of the crystal structures of a range of adducts of SeF_2_(CN)_2_ with several oxygen- and nitrogen-bearing molecules is dominated by the presence of multiple Se^IV^∙∙∙O/N ChBs. Ibrahim and coworkers [55] conducted a comparative study concerning the σ-hole and lone-pair-hole site-based interactions of YF_4_ (Y = S, Se) compounds with NH_3_ and HF Lewis bases. Their findings disclosed that the σ-hole interactions are significantly stronger than the lone-pair-hole interactions. For the same Lewis base, the ChB distance decreases and the binding energy becomes more negative from SF_4_ to SeF_4_. Recently, we computationally and comparatively investigated the heterodimers of SeO_2_ with various O-/S-bearing Lewis bases [56]. The results suggested that the binding distances between Se and O atoms range from 2.512 to 2.765 Å, and the binding distances between Se and S atoms span from 2.818 to 3.309 Å. These π-hole-type Se∙∙∙O/S ChBs are notably strong and exhibit a certain degree of covalent character in most cases. The interaction energies of these chalcogen-bonded heterodimers are in the range of −3.53 to −13.77 kcal/mol.

Additionally, it has also been evidenced that the strength of ChBs can be influenced by the different valencies of electron-deficient atoms in Lewis acids and the hybridization effects of electron-rich atoms in Lewis bases [34,42,57]. For instance, Scheiner’s group [42] comparatively studied the S∙∙∙π ChBs between SF_2_/SF_4_ and a range of unsaturated hydrocarbons, and the results revealed that the S^II^∙∙∙π ChBs in the SF_2_ heterodimers are generally shorter and stronger than the S^IV^∙∙∙π ChBs in the SF_4_ heterodimers, except for SF_4_∙∙∙benzene. This group also theoretically characterized the S∙∙∙N ChBs in the heterodimers of SF_4_ with a suite of alkylamines (sp^3^) and nitrogen-containing heteroaromatic amines (sp^2^) [34]. It was disclosed that these S∙∙∙N ChBs have substantial binding strength, with the corresponding binding energies ranging from −6.62 kcal/mol for NH_3_ to −14.39 kcal/mol for N(CH_3_)_3_. Remarkably, the S∙∙∙N ChBs in the heterodimers containing heteroaromatic amines are weaker than those in all the heterodimers containing alkylamines, with the exception of NH_3_.

Herein, the current study aimed to address the existing gaps in our understanding of selenium-centered ChBs. We can gain direct theoretical insights into the differential effects of divalency and tetravalency through a comparison of SeF_2_ with SeF_4_. A series of different oxygen-bearing molecules, including those with sp^3^-hybridization (H_2_O, CH_3_OH, CH_3_OCH_3_, and ethylene oxide (C_2_H_4_O)) and those with sp^2^-hybridization (HCHO, CH_3_CHO, CH_3_COCH_3_, and HCOOH), were chosen as Lewis bases to participate in the Se^II/IV^∙∙∙O ChBs by interacting with SeF_2_ and SeF_4_, respectively. Moreover, several computational tools, including molecular electrostatic potential (MEP) surfaces [58], quantum theory of atoms in molecules (QTAIM) [59], non-covalent interaction plot (NCIplot) [60], natural bond orbital (NBO) [61], and symmetry-adapted perturbation theory (SAPT) [62] approaches were utilized to comprehensively characterize the strength and nature of the Se^II/IV^∙∙∙O ChBs. Concurrently, in addition to examining the effects of valency and hybridization on the Se∙∙∙O ChBs in terms of the strength and nature, we also carried out a comparative analysis of the σ-hole-type Se∙∙∙O ChBs reported in this study with the π-hole-type Se∙∙∙O ChBs observed in the SeO_2_ heterodimers [56].

## 2. Results and Discussion

### 2.1. MEP Analyses for the Monomers

**Figure 1:**
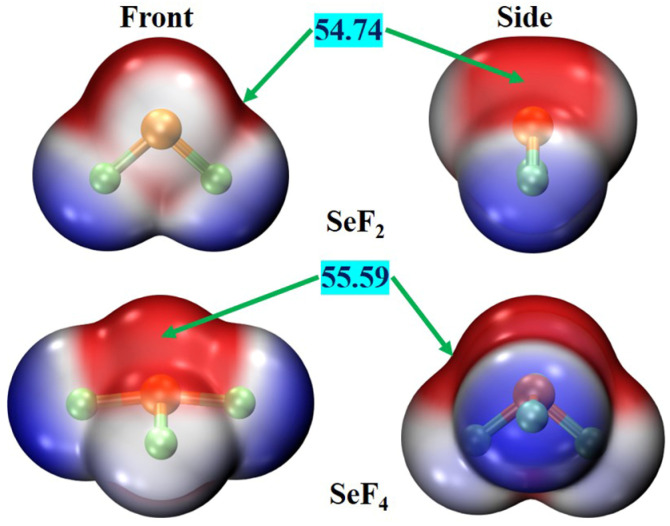
MEP maps of SeF_2_ and SeF_4_ on the 0.001 electron/Bohr^3^ isosurface. The blue and red areas correspond to the negative and positive electrostatic potentials, respectively. The *V*_s,max_ values are reported in kcal/mol.

The MEP has been proven to be an effective analytical tool to predict and evaluate intermolecular interactions by identifying the electrophilic and nucleophilic sites within a molecule [58]. Therefore, MEP analyses were conducted for two Lewis acids and eight oxygen-containing Lewis bases. Figure 1 illustrates the MEPs of the SeF_2_ and SeF_4_ monomers. There are two positive electrostatic potential regions (red areas) positioned along the extension of the F–Se bonds for both SeF_2_ and SeF_4_. These red areas around the Se atoms represent the σ-holes. The most positive MEP values (*V*_s,max_) on the holes are 54.74 kcal/mol for SeF_2_ and 55.59 kcal/mol for SeF_4_. It is readily apparent that the magnitude of this hole is largely unaffected by the valency of the Se atom. It should be pointed out that although SeF_4_ has four F–Se bonds, no σ-holes exist along the axial F–Se bond extension. This can be attributed to the fact that the axial F atoms are directly opposed to one another. For the oxygen-containing Lewis bases, there are the blue regions surrounding the O atoms, which correspond to the negative electrostatic potential areas, as displayed in Figure S1 in the Supplementary Materials (SM). The computed most negative MEP values (*V*_s,min_) associated with the O atoms are in the range of −34.39 to −42.01 kcal/mol (see Table 1). The *V*_s,min_ value becomes more negative in the following order: CH_3_OCH_3_ < HCOOH < HCHO < H_2_O < CH_3_OH < C_2_H_4_O < CH_3_CHO < CH_3_COCH_3_. It is notable that HCOOH has the negative electrostatic potential regions around its two O atoms, where the electrostatic potential of the carbonyl O atom is significantly more negative than that of the hydroxyl O atom (see Figure S1). Furthermore, these oxygen-containing compounds have positive electrostatic potential regions (red areas) around the H atoms. Consequently, the Se atoms in both SeF_2_ and SeF_4_ were expected to interact with the O atoms in eight oxygen-bearing compounds, leading to the formation of the Se∙∙∙O ChBs.

### 2.2. Structural and Energetic Characteristics of the Heterodimers

**Figure 2:**
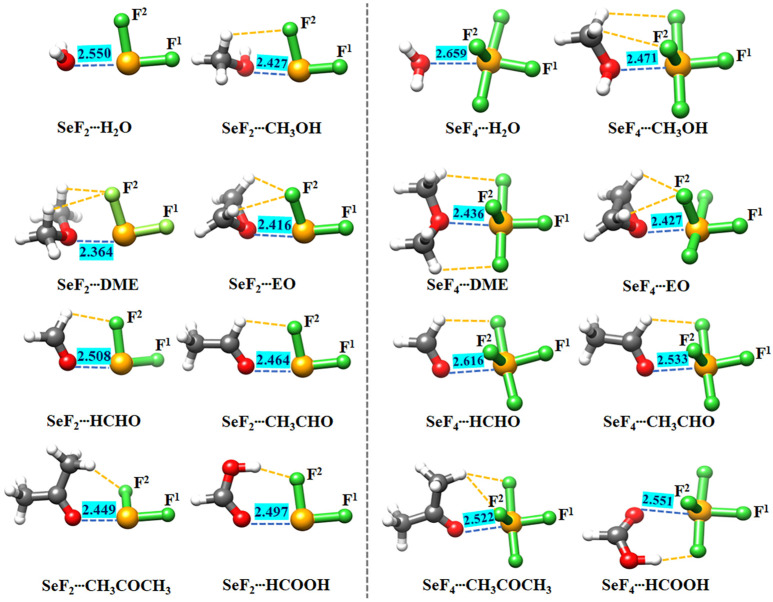
The optimized geometrical structures of the examined heterodimers, marked with the *R*(Se∙∙∙O) distance (Å). The Se∙∙∙O ChBs and C/O–H∙∙∙F HBs uncovered using the NCIplot approach are depicted by the blue and orange dotted lines, respectively.

The global minimum structure for each of the heterodimers of SeF_2_ and SeF_4_ with eight oxygen-bearing Lewis bases is displayed in Figure 2, and their Cartesian coordinates are collected in Table S1 in the Supplementary Materials. The key structural parameters of the examined heterodimers are reported in Table 2. As shown in Figure 2, the structures exhibiting C_s_ symmetry are the heterodimers of SeF_2_ with H_2_O, CH_3_OCH_3_, C_2_H_4_O, HCHO, CH_3_CHO, and HCOOH, and the heterodimers of SeF_4_ with CH_3_OCH_3_ and C_2_H_4_O. Conversely, the other heterodimers have C_1_ symmetry. The O atom is situated toward the Se atom along the F^1^–Se bond extension of both SeF_2_ and SeF_4_ subunits in all the studied heterodimers. It is evidenced that the *R*(Se∙∙∙O) distances range from 2.364 Å to 2.659 Å, which are markedly lower than the sum of the van der Waals radii of two interacting atoms (3.42 Å) by 22.3% to 30.9% [63], thus suggesting the formation of a strong Se∙∙∙O ChB within each heterodimer. The corresponding ∠F^1^–Se∙∙∙O angle varies between 164.1° and 174.1°. The observed deviation from linearity is a consequence of the presence of the H∙∙∙F hydrogen bonds. For the same oxygen-containing Lewis base, the Se^II^∙∙∙O distances in the SeF_2_ heterodimers are significantly shorter than the Se^IV^∙∙∙O distances in the SeF_4_ and SeO_2_ [56] heterodimers. This suggests that the Se^II^∙∙∙O ChBs are stronger than the σ- and π-hole-type Se^IV^∙∙∙O ChBs. However, these Se^II/IV^∙∙∙O distances are all clearly smaller than the σ-hole-type Se^II^∙∙∙O ChBs observed in proteins [27] protein-ligand complexes [47,64], and binary clusters [65]. For the fixed Lewis acid, the *R*(Se∙∙∙O) distances in the heterodimers containing sp^2^-hybridized oxygen bases are longer than those in the heterodimers containing sp^3^-hybridized oxygen bases, with the exception of H_2_O. Simultaneously, it is important to highlight that as the methyl group is gradually introduced, there is a notable reduction in the *R*(Se∙∙∙O) distance in the heterodimers of SeF_2_ and SeF_4_ with CH_3_OR and CH_3_COR (R = H, CH_3_) in comparison to the heterodimers involving H_2_O and HCHO. Moreover, the presence of a hydroxyl group in both SeF_2_∙∙∙HCOOH and SeF_4_∙∙∙HCOOH heterodimers also leads to a reduction in the *R*(Se∙∙∙O) distance by 0.011 Å and 0.065 Å, respectively, compared with the SeF_2_∙∙∙HCHO and SeF_4_∙∙∙HCHO heterodimers. This phenomenon can be attributed to the electron-donating properties of the methyl and hydroxyl groups, which can facilitate the electron donation from the oxygen-containing Lewis bases.

Table 2 also presents the variations in the internal bond lengths that occur during the formation of the investigated heterodimers. It is observed that the Se–F bond lengths of both SeF_2_ and SeF_4_ subunits in the heterodimers are stretched in comparison to the isolated SeF_2_ and SeF_4_ monomers. The Se–F^1^ bond that is directly opposite the O atom elongates by 0.015 to 0.030 Å for SeF_2_ and by 0.007 to 0.018 Å for SeF_4_. Generally, the larger the elongation of the Se–F^1^ bond length, the shorter the corresponding Se∙∙∙O distance. For the Se–F^2^ bond, which corresponds to another Se–F bond of SeF_2_ and another equatorial Se–F bond of SeF_4_, less stretching is found in all the heterodimers studied, except for the SeF_2_∙∙∙HCOOH and SeF_4_∙∙∙HCOOH heterodimers. Compared to the Se–F^1^ bond, there is a larger elongation for the Se–F^2^ bond in the SeF_2_∙∙∙HCOOH heterodimer, while a smaller contraction for the Se–F^2^ bond in the SeF_4_∙∙∙HCOOH heterodimer is observed. These elongations partly result from the charge transfer from the Lewis bases to the σ*(Se–F) antibonding orbitals (as discussed below). Similar elongations were also documented in the heterodimers containing SHF, SF_2_, or SF_4_ [34,42,43,55].

Additionally, the interaction energies (*E*_int_), binding energies (*E*_b_), and deformation energy (*E*_def_) of the examined heterodimers were computed and collected in the other columns of Table 2. It should be pointed out that the *E*_def_, which represents the difference between the *E*_b_ and the *E*_int_, was utilized to evaluate the degree of distortion of the two subunits in each heterodimer. One can see that the *E*_int_ values range from −5.25 to −9.76 kcal/mol for the SeF_2_ heterodimers and from −5.42 to −11.16 kcal/mol for the SeF_4_ heterodimers. It should be pointed out that the *E*_int_ values are generally in accordance with the interaction energies computed at the CCSD(T)/CBS level of theory for all the studied heterodimers, confirming the accuracy and reliability of MP2/aug-cc-pVTZ level. The *E*_def_ values of the SeF_2_ and SeF_4_ heterodimers are all clearly smaller than those of the SeO_2_ heterodimers [56] when the same oxygen-containing Lewis base is considered. The largest *E*_def_ values are observed for the SeF_2_∙∙∙HCOOH and SeF_4_∙∙∙HCOOH heterodimers, approaching 1.31 and 2.69 kcal/mol, respectively. This indicates that the formation of a strong O–H∙∙∙F hydrogen bond in each of the two heterodimers (see below) results in a significant distortion of the monomeric structures. The consideration of the deformation energy leads to a reduction of the absolute *E*_b_ values to a range of 5.07–8.82 kcal/mol for the SeF_2_ heterodimers and 5.04–8.54 kcal/mol for the SeF_4_ heterodimers. For a given oxygen-containing Lewis base, the *E*_int_ values for the SeF_2_ heterodimers are less negative than those for the SeF_4_ heterodimers, whereas the *E*_b_ values exhibit the reverse trend. It is worth noting that the *E*_b_ values for both SeF_2_ and SeF_4_ heterodimers are more negative than those for the SeO_2_ heterodimers [56]. The former involves the σ-hole-type Se∙∙∙O ChBs, while the latter involves the π-hole-type Se∙∙∙O ChBs. These findings demonstrate that the order of the electron-accepting ability is SeF_2_ > SeF_4_ > SeO_2_. Furthermore, the results reveal that the introduction of the methyl and hydroxyl groups can significantly increase both the interaction and binding energies. It is noteworthy that the observed increase in the interaction and binding energies of these heterodimers is accompanied by a decrease in the corresponding Se∙∙∙O distances.

### 2.3. QTAIM Analyses

Since Bader’s QTAIM method [59] is a frequently used analytical technique for estimating and understanding the strength and nature of various non-covalent interactions in terms of topology analysis of the electron density, QTAIM analyses for the sixteen heterodimers were performed. As illustrated in Figure S2, the QTAIM analysis findings disclose the existence of a Se∙∙∙O contact because there is a bond critical point (BCP, indicated by an orange dot) and a bond path (BP, indicated by a brown line) connecting two chalcogen atoms in each heterodimer. The BCPs and BPs between the F atoms in Lewis acids and the H atoms in oxygen-bearing Lewis bases were not identified for the SeF_2_∙∙∙H_2_O, SeF_2_∙∙∙CH_3_OH, SeF_2_∙∙∙CH_3_OCH_3_, SeF_2_∙∙∙C_2_H_4_O, SeF_4_∙∙∙H_2_O, and SeF_4_∙∙∙C_2_H_4_O heterodimers, suggesting the absence of the H∙∙∙F HBs. Conversely, all other heterodimers have one or two BCPs and BPs related to the H∙∙∙F HBs.

**Figure 3:**
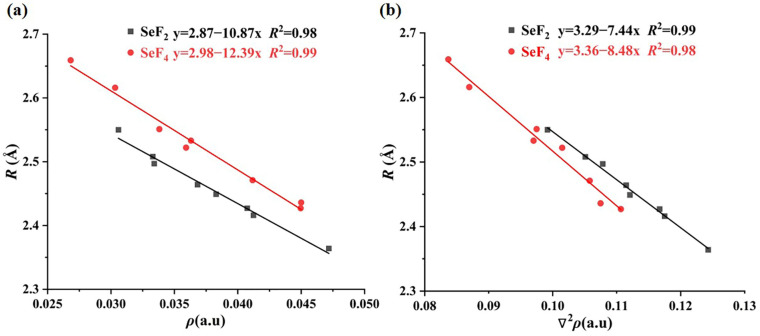
Correlation plots of the Se∙∙∙O distance (*R*) versus the electron density (*ρ*, (**a**)) and its Laplacian (∇^2^*ρ*, (**b**)) at the Se∙∙∙O BCP.

Table 3 presents the computed topological parameters at the Se∙∙∙O and H∙∙∙F BCPs. These parameters include the electron density (*ρ*), Laplacian of electron density (∇^2^*ρ*), potential energy density (*V*), kinetic energy density (*G*), and total energy density (*H*). Simultaneously, the absolute ratio between *G* and *V* was also computed and is reported in the last column of Table 3. One can find that the *ρ* values at the Se∙∙∙O BCPs range from 0.0306 to 0.0472 a.u for the SeF_2_ heterodimers and from 0.0268 to 0.0450 a.u for the SeF_4_ heterodimers. The ∇^2^*ρ* values vary between 0.0992 and 0.1243 a.u for the SeF_2_ heterodimers and between 0.0837 and 0.1107 a.u for the SeF_4_ heterodimers, respectively. Both *ρ* and ∇^2^*ρ* exhibit a good linear relationship with the Se∙∙∙O distance, as evidenced by the correlation coefficient ranging from 0.98 to 0.99 for the SeF_2_ and SeF_4_ heterodimers in Figure 3. For the fixed oxygen-bearing Lewis base, both *ρ* and ∇^2^*ρ* values at the Se∙∙∙O BCP for each of the SeF_2_ and SeF_4_ heterodimers are clearly larger than those for each SeO_2_ heterodimer [56]. This demonstrates that the strength of the σ-hole-type Se∙∙∙O ChBs is stronger than that of the π-hole-type Se∙∙∙O ChBs. Both the Se^II^∙∙∙O and Se^IV^∙∙∙O ChBs in the inspected heterodimers are closed-shell interactions, as evidenced by the existence of positive ∇^2^*ρ* values. Furthermore, the Se^IV^∙∙∙O ChB in the SeF_4_∙∙∙H_2_O heterodimer belongs to the non-covalent nature due to the presence of *H* > 0 and |*G*/*V*| > 1, while the Se^II/IV^∙∙∙O ChBs in the remaining heterodimers exhibit a partially covalent nature, as indicated by the values of *H* < 0 and 0.5 < |*G*/*V*| < 1. It is important to note that the topological properties of the Se∙∙∙O ChBs are influenced by the presence of methyl and hydroxyl groups, as well as by the hybridization state of the oxygen atom in Lewis bases. These effects align with the trends observed in the Se∙∙∙O distances, interaction energies and binding energies, as detailed in Table 2. Additionally, it can be observed that the *ρ* and ∇^2^*ρ* values at the H∙∙∙F BCPs are typically less than 0.02 and 0.05 a.u, respectively. The exceptions are found for the SeF_2_∙∙∙HCOOH and SeF_4_∙∙∙HCOOH heterodimers, where the largest values for both *ρ* and ∇^2^*ρ* are observed, accompanied by the negative values for *H*. This evidence indicates that these two H∙∙∙F HBs are extremely strong and possess partial covalency.

We also quantitatively evaluated the energy (*E*_NCI_) of individual Se∙∙∙O ChBs and H∙∙∙F HBs identified by the QTAIM analyses according to the following two formulas [66,67]:

*E*_NCI_ = 0.375 × *V* − 0.5655 (for the Se∙∙∙O ChBs) (1)

*E*_NCI_ = −223.08 × *ρ* + 0.7423 (for the H∙∙∙F HBs) (2)

One can see from Table 3 that the *E*_NCI_ value for each Se^II^∙∙∙O ChB falls within the range of −6.57 to −10.97 kcal/mol, and it varies between −5.24 and −9.47 kcal/mol for each Se^IV^∙∙∙O ChB. Remarkably, for the same oxygen-containing Lewis base, almost all the Se^II^∙∙∙O ChBs have higher *E*_NCI_ values than the Se^IV^∙∙∙O ChBs. The exceptions are observed for the heterodimers containing C_2_H_4_O, where the Se^II^∙∙∙O ChBs have a marginally smaller *E*_NCI_ value than the Se^IV^∙∙∙O ChBs. It was also found that the H∙∙∙F HBs in the SeF_2_∙∙∙HCOOH and SeF_4_∙∙∙HCOOH heterodimers have *E*_NCI_ values of −5.63 and −7.38 kcal/mol, respectively. Interestingly, the H∙∙∙F HB shows a larger *E*_NCI_ value than the Se^IV^∙∙∙O ChB in the SeF_4_∙∙∙HCOOH heterodimer, indicating that the H∙∙∙F HB is stronger than the Se^IV^∙∙∙O ChB.

### 2.4. NCIplot Analyses

**Figure 4:**
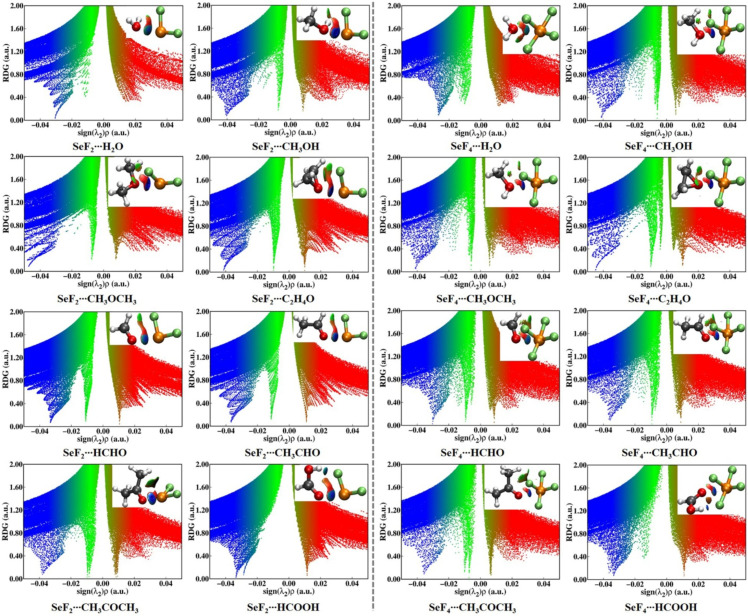
RDG isosurfaces (*s* = 0.55 a.u) and scatter plots of RDG versus sign(λ_2_)*ρ* for the investigated heterodimers. The weak and strong attractive interactions are represented by the green- and blue-colored isosurfaces, respectively, and the repulsive interactions are indicated by the red-colored isosurfaces.

The application of the NCIplot method [60] allowed further understanding of the nature of the intermolecular interactions in the investigated heterodimers. Figure 4 illustrates the diagrams of reduced density gradient (RDG) isosurfaces for the studied heterodimers. The weak and strong attractive interactions are indicated by the green- and blue-colored areas, respectively, while the red-colored areas correspond to the repulsive interactions. Apparently, a blue-colored isosurface is present between two chalcogen atoms in each heterodimer, which further corroborates the presence of a strongly attractive ChB. In the SeF_2_∙∙∙H_2_O and SeF_4_∙∙∙H_2_O heterodimers, no green-colored isosurfaces are present between the F atoms of SeF_2_/SeF_4_ and the H atoms of H_2_O. However, one or two green-/blue-colored isosurfaces are observed between the H and F atoms in the other heterodimers. Interestingly, the H∙∙∙F HBs, which were undetected by the QTAIM analyses, were identified by the presence of green-colored isosurfaces in the NCIplot diagrams of the SeF_2_∙∙∙CH_3_OH, SeF_2_∙∙∙CH_3_OCH_3_, SeF_2_∙∙∙C_2_H_4_O, and SeF_4_∙∙∙C_2_H_4_O heterodimers.

Figure 4 also depicts the scatter plots of RDG versus the product of the sign of the second eigenvalue λ_2_ and the electron density (*ρ*) for the studied heterodimers. It is crucial to note that attractive interactions are characterized by a negative value of sign(λ_2_)*ρ*, while repulsive interactions are indicated by a positive value of sign(λ_2_)*ρ*. The analysis revealed that the peaks associated with the Se∙∙∙O ChBs and H∙∙∙F HBs are consistently situated at negative sign(λ_2_)*ρ* values, thereby substantiating the presence of attractive non-covalent interactions. Moreover, for a given Lewis base with the exception of CH_3_OH and C_2_H_4_O, the Se^II^∙∙∙O ChBs in the SeF_2_ heterodimers have a more negative sign(λ_2_)*ρ* value than the Se^IV^∙∙∙O ChBs in the SeF_4_ heterodimers, demonstrating that most Se^II^∙∙∙O ChBs are stronger than the Se^IV^∙∙∙O ChBs. However, all the Se∙∙∙O ChBs exhibit a more negative sign(λ_2_)*ρ* value than the H∙∙∙F HBs within each heterodimer, except for the SeF_4_∙∙∙HCOOH heterodimer. In the latter case, the peak associated with the H∙∙∙F HB is positioned at a more negative sign(λ_2_)*ρ* value than the Se∙∙∙O ChB, indicating that the latter is weaker than the former. Noticeably, the presence of methyl and hydroxyl groups in the heterodimers of SeF_2_ and SeF_4_ with HCOOH, CH_3_OR, and CH_3_COR (R = H, CH_3_) markedly diminishes the absolute value of sign(λ_2_)*ρ* in comparison to the heterodimers comprising HCHO and H_2_O. Concurrently, it is noteworthy that for the fixed Lewis acid, the sp^2^-hybridized oxygen bases exhibit less negative sign(λ_2_)*ρ* values than those for the sp^3^-hybridized oxygen bases, with the exception of H_2_O. These findings are consistent with those obtained in the QTAIM analyses.

### 2.5. NBO Analyses

**Figure 5:**
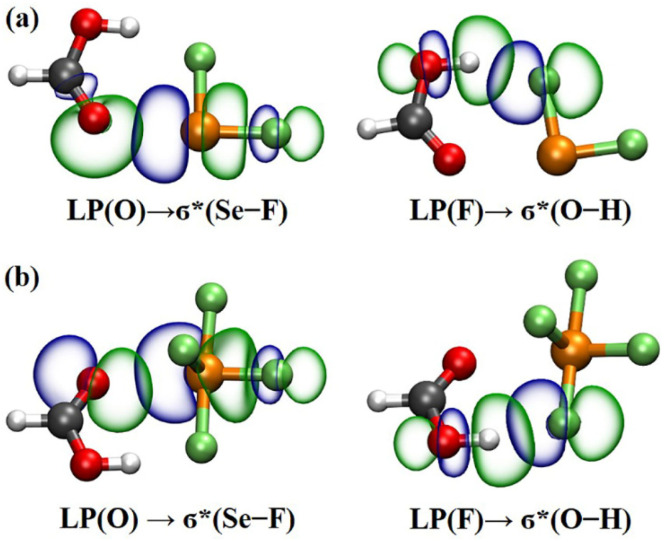
Plots of the NBOs in both SeF_2_∙∙∙HCOOH (**a**) and SeF_4_∙∙∙HCOOH (**b**) heterodimers related to the Se∙∙∙O ChBs and H∙∙∙F HBs.

The implementation of NBO analyses [61] provided a valuable additional perspective on the nature of the Se∙∙∙O ChBs through the examination of orbital interactions and the corresponding second-order perturbation energies (*E*^(2)^). Table 4 shows the orbital interactions related to the Se∙∙∙O ChBs and their corresponding *E*^(2)^ values. Generally, the larger the *E*^(2)^ value associated with orbital interaction, the stronger the corresponding chalcogen bonding interaction. The findings reveal that the primary stabilization of the majority of the studied heterodimers arises from the interactions of the lone pair (LP) of the O atom in Lewis bases with the σ*(Se–F^1^) antibonding orbitals of SeF_2_ and SeF_4_. The LP(O) → σ*(Se–F^1^) orbital interactions have the *E*^(2)^ values ranging from 11.52 to 18.12 kcal/mol for the SeF_2_ heterodimers and from 5.82 to 13.93 kcal/mol for the SeF_4_ heterodimers. The results further demonstrate that strong σ-hole-type Se∙∙∙O ChBs are formed, as evidenced by a substantial overlap between the LP(O) of Lewis base and the σ*(Se–F^1^) antibonding orbital of SeF_2_/SeF_4_ in each heterodimer (see Figure S3). It is interesting to emphasize that an additional interaction between the LP(O) of Lewis base and the σ*(Se–F^2^) antibonding orbital of SeF_2_/SeF_4_ was also observed in each heterodimer, apart from the LP(O) → σ*(Se–F^1^) interaction. However, the *E*^(2)^ values for the LP(O) → σ*(Se–F^2^) interactions are significantly smaller than those for the LP(O) → σ*(Se–F^1^) interactions. Moreover, we also observed the back-transfer of charge from the SeF_2_/SeF_4_ subunit to the oxygen-containing Lewis base. Specifically, the charge transfer occurs from the LP(Se) into the σ*(O–H), σ*(O–C), or π*(O=C) antibonding orbitals of Lewis bases. The *E*^(2)^ values of these orbital interactions are all less than 1 kcal/mol. Notably, there is a markedly strong interaction between the LP(F) and the σ*(O–H) antibonding orbital in the SeF_2_∙∙∙HCOOH and SeF_4_∙∙∙HCOOH heterodimers, as represented by a significant overlap between the two orbitals (see Figure 5). The *E*^(2)^ values for the LP(F) → σ*(O–H) interactions are 9.96 kcal/mol in the SeF_2_∙∙∙HCOOH heterodimer and 15.34 kcal/mol in the SeF_4_∙∙∙HCOOH heterodimer. This suggests that the H∙∙∙F HB is clearly weaker in strength than the Se^II^∙∙∙O ChB in the former but significantly stronger than the Se^IV^∙∙∙O ChB in the latter. Finally, it is noteworthy that the valence of the Se atom in Lewis acids, the introduction of electron-donating methyl and hydroxyl groups, and the hybridization type of the O atom in Lewis bases also have a significant influence on the *E*^(2)^ values of the orbital interactions related to the Se∙∙∙O ChBs. For example, the *E*^(2)^ values related to the Se^II^∙∙∙O ChBs in the SeF_2_ heterodimers are markedly higher than those pertaining to the Se^IV^∙∙∙O ChBs in the SeF_4_ heterodimers when the same oxygen-containing Lewis base is considered. For the fixed Lewis acid, the *E*^(2)^ values gradually increases with the order of H_2_O(HCHO) < CH_3_OH(CH_3_CHO) < CH_3_OCH_3_(CH_3_COCH_3_). Overall, the observed trend in the *E*^(2)^ values qualitatively aligns with the previously discussed *E*_int_ values and NCIplot results.

### 2.6. SAPT Analyses

**Figure 6:**
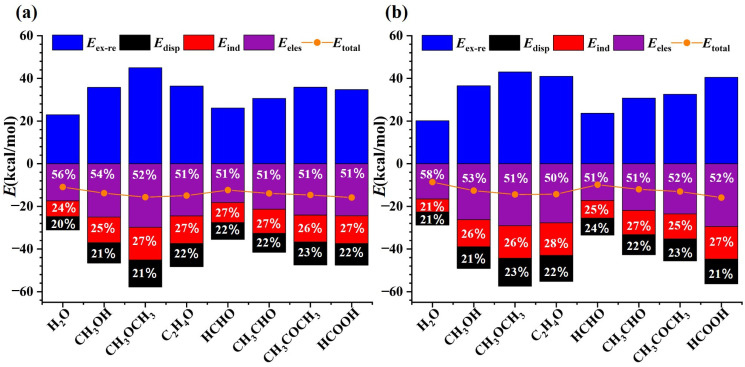
Summary of the attractive and repulsive components for the SeF_2_ (**a**) and SeF_4_ (**b**) heterodimers. The interpolated values represent the percentage contribution of each attractive component to the total attractive energy of each heterodimer.

In order to obtain deeper insight into the intrinsic nature of the non-covalent interactions between two interacting subunits from a quantitative perspective, SAPT analyses [62] were also conducted for both SeF_2_ and SeF_4_ heterodimers. This approach allows to disentangle the total interaction energies (*E*_total_) of the studied heterodimers into four physically meaningful components, including electrostatic (*E*_elec_), induction (*E*_ind_), dispersion (*E*_disp_), and exchange–repulsion (*E*_ex-re_). The SAPT analysis findings are tabulated in Table S2 and are graphically present in Figure 6. Apparently, the results suggest that the electrostatic component constitutes the predominant attractive force in stabilizing the examined heterodimers, and its contribution accounts for approximately 51–56% and 50–58% of the total attractive interaction energies for the SeF_2_ and SeF_4_ heterodimers, respectively. Furthermore, the induction contribution is slightly larger than the dispersion one in all the investigated heterodimers, with the exception of SeF_4_∙∙∙H_2_O. In this case, the induction and dispersion components contribute equally. However, the combined contributions from both induction and dispersion components (42–50%) are also significant and cannot be disregarded. Noticeably, the electrostatic contribution progressively decreases with the increasing addition of methyl groups in the SeF_2_∙∙∙CH_3_OR and SeF_4_∙∙∙CH_3_COR heterodimers (R = H, CH_3_) in comparison with the SeF_2_/SeF_4_∙∙∙H_2_O heterodimers. Conversely, the variation in the number of methyl groups exhibits minimal impact on the electrostatic contribution in the SeF_2_∙∙∙CH_3_OR and SeF_4_∙∙∙CH_3_COR heterodimers (R = H, CH_3_) relative to the SeF_2_/SeF_4_∙∙∙HCHO heterodimers. Moreover, the introduction of a hydroxyl group has no effect on the contribution of each attractive component in the SeF_2_∙∙∙HCOOH heterodimer, although it does exert a certain effect on the contribution of each attractive component in the SeF_4_∙∙∙HCOOH heterodimer, compared to the SeF_2_∙∙∙HCHO and SeF_4_∙∙∙HCHO heterodimers. For the fixed Lewis base, the valency state of Se atoms has also little effect on the contribution of each attractive component.

It is also estimated that the total interaction energies range from −8.11 to −12.84 kcal/mol for the SeF_2_ heterodimers and from −8.63 to −15.90 kcal/mol for the SeF_4_ heterodimers. For the same oxygen base, the *E*_total_ value becomes more negative in the order of SeF_2_ < SeF_4_. This trend matches the *E*_int_ values reported in Table 2, as illustrated by a strong linear relationship in Figure S4, with a correlation coefficient of 0.99.

## 3. Computational Methods

The MP2/aug-cc-pVTZ computational level [68,69] was employed to fully optimize the geometric structures of the isolated monomers and their heterodimers. The high accuracy and reliability of this level have been proven in studying chalcogen bonding interactions [55,56,57,70,71]. Frequency analysis was also conducted at the same level to confirm that all the obtained geometric structures are real minima with positive vibrational frequencies and to calculate the zero-point vibrational energies (ZPE). The interaction energies (*E*_int_) and binding energies (*E*_b_) of the studied heterodimers were evaluated using the following equations:

*E*_int_ = *E*_AB_ − *E*_A_ − *E*_B_ (3)

*E*_b_ = *E*_AB_ − *E*_A’_ − *E*_B’_ (4)

where *E*_AB_ indicates the total energy of the optimized heterodimer, *E*_A_ and *E*_B_ denote the total energies of the monomers adopted in the heterodimer, and *E*_A’_ and *E*_B’_ represent the total energies of the isolated optimized monomers. The ZPE and BSSE (basis set superposition error) [72] corrections were also considered for all these quantities. The CCSD(T)/CBS level was also employed to calculate the interaction energies of all the examined heterodimers to validate the accuracy and reliability of the MP2/aug-cc-pVTZ level using the following equations [73,74]:

*E*_CCSD(T)/CBS_ = Δ*E*_MP2/CBS_ + (Δ*E*_CCSD(T)/aug-cc-pVDZ_ − Δ*E*_MP2/aug-cc-pVDZ_) (5)

Δ*E*_MP2/CBS_ = (64*E*_MP2/aug-cc-pVQZ_ − 27*E*_MP2/aug-cc-pVTZ_)/37 (6)

All the calculations mentioned above were implemented employing the Gaussian 16 program [75].

The MEP surface of each monomer on the 0.001 electron/Bohr^3^ isosurface was calculated at the same level with the Multiwfn program [76]. Based on the MP2/aug-cc-pVTV- level-optimized geometries of the studied heterodimers, the QTAIM and NCIPlot analyses were conducted using the Multiwfn program. The B3LYP-D3(BJ)/aug-cc-pVTZ level was chosen to perform the NBO analysis [61] in the NBO 7.0 program [77]. The MEP, QTAIM, NCIPlot, and NBO analysis results were visually represented by means of the VMD software (version 1.9.3) [78]. The PSI4 software (version 1.3.2) [79] was employed to implement the SAPT analysis at the SAPT2+3/aug-cc-pVTZ level [62].

## 4. Conclusions

In summary, we conducted ab initio computations to investigate Se∙∙∙O chalcogen bonding interactions in the model heterodimers of SeF_2_ and SeF_4_ with a range of oxygen-bearing Lewis bases. These Se∙∙∙O contacts were comprehensively analyzed by means of the MEP, QTAIM, NCIplot, NBO, and SAPT methodologies in terms of strength and nature. The MEP analysis revealed that the σ-hole regions are located on the Se atom of both SeF_2_ and SeF_4_ molecules, which form the σ-hole-type Se∙∙∙O interactions with the nucleophilic regions of Lewis bases on the O atoms. These Se∙∙∙O distances are relatively short, varying from 2.364 Å to 2.659 Å, compared with the sum of the van der Waals radii of two chalcogen atoms. Upon the formation of each chalcogen bonding interaction, the internal Se–F covalent bond lengths of both SeF_2_ and SeF_4_ subunits in the heterodimers are observed to undergo a general elongation in comparison with the isolated SeF_2_ and SeF_4_ monomers. The interaction energies fall within the range of −5.25 to −11.16 kcal/mol. The Se∙∙∙O ChBs belong to the non-covalent closed-shell interactions, exhibiting a certain degree of covalent character in most cases. For the fixed Lewis base, the Se^II^∙∙∙O ChBs are stronger in the SeF_2_ heterodimers than the Se^IV^∙∙∙O ChBs in the SeF_4_ heterodimers, as demonstrated by the QTAIM, NCIplot, and NBO approaches. The NBO analysis results also indicated that the LP(O) → σ*(Se–F^1^) orbital interactions predominantly stabilize all the examined heterodimers, with the exception of SeF_4_∙∙∙HCOOH, where the LP(F) → σ*(O–H) orbital interaction was found to be the primary stabilizing factor. The presence of electron-donating methyl and hydroxyl groups can significantly strengthen such ChBs. Additionally, the utilization of the SAPT method showed that the electrostatic component is the most significant attractive force, accounting for more than half of the total stabilization of the targeted heterodimers. However, it is also necessary to consider the contributions of both induction and dispersion components. Notably, the *E*_total_ obtained from the SAPT analyses of the studied heterodimers exhibited a highly significant linear correlation with the corresponding interaction energies. Overall, these observations will contribute to improving our comprehension of the influence of valency state, hybridization type, and electron-donating groups on chalcogen bonding interactions and may lay the foundation for forthcoming investigations associated with materials science, catalysis, and crystal engineering.

## Figures and Tables

**Table 1:** The *V*_s,max_ and *V*_s,min_ values (kcal/mol) on the 0.001 electron/Bohr^3^ isosurface computed at the MP2/aug-cc-pVTZ level for all the involved monomers.

Monomer	*V*_s,min_	Monomer	*V*_s,min_	Monomer	*V*_s,max_
H_2_O	−35.65	HCHO	−34.97	SeF_2_	54.74
CH_3_OH	−36.09	CH_3_CHO	−39.31	SeF_4_	55.59
CH_3_OCH_3_	−34.39	CH_3_COCH_3_	−42.01		
C_2_H_4_O	−36.18	HCOOH	−34.51 ^a^		

^a^ This value corresponds to the carbonyl oxygen atom.

**Table 2:** The *R*(Se∙∙∙O) distance (Å), F^1^–Se∙∙∙O angles (°), change in Se–F^1/2^ bond lengths (Δ*R*, Å) between the monomer and the heterodimer, interaction energy (*E*_int_, kcal/mol), binding energy (*E*_b_, kcal/mol), and deformation energy (*E*_def_, kcal/mol) within the studied heterodimers.

Heterodimer	*R*(Se∙∙∙O)	∠F^1^–Se∙∙∙O	Δ*R*(Se–F^1^)	Δ*R*(Se–F^2^)	*E*_int_	*E*_b_	*E*_def_
SeF_2_∙∙∙H_2_O	2.550(25.4%) ^a^	174.0	0.0173	0.0131	−5.25(−6.16) ^b^	−5.07	0.18
SeF_2_∙∙∙CH_3_OH	2.427(29.0%)	172.7	0.0248	0.0174	−8.11(−8.41)	−7.43	0.67
SeF_2_∙∙∙CH_3_OCH_3_	2.364(30.9%)	172.4	0.0304	0.0194	−9.76(−9.23)	−8.82	0.94
SeF_2_∙∙∙C_2_H_4_O	2.416(29.4%)	173.1	0.0255	0.0203	−9.20(−9.10)	−8.52	0.67
SeF_2_∙∙∙HCHO	2.508(26.7%)	173.3	0.0191	0.0162	−6.58(−7.19)	−6.10	0.48
SeF_2_∙∙∙CH_3_CHO	2.464(28.0%)	173.7	0.0236	0.0173	−8.10(−8.21)	−7.43	0.67
SeF_2_∙∙∙CH_3_COCH_3_	2.449(28.4%)	173.1	0.0267	0.0153	−8.55(−8.22)	−7.48	1.07
SeF_2_∙∙∙HCOOH	2.497(27.0%)	174.1	0.0146	0.0371	−9.18(−9.87)	−7.87	1.31
SeF_4_∙∙∙H_2_O	2.659(22.3%)	164.1	0.0066	0.0033	−5.42(−5.93)	−5.04	0.38
SeF_4_∙∙∙CH_3_OH	2.471(27.7%)	171.1	0.0143	0.0099	−8.96(−8.21)	−7.11	1.85
SeF_4_∙∙∙CH_3_OCH_3_	2.436(28.8%)	170.8	0.0176	0.0114	−10.55(−8.96)	−8.54	2.01
SeF_4_∙∙∙C_2_H_4_O	2.427(29.0%)	171.8	0.0169	0.0163	−10.39(−9.38)	−8.34	2.05
SeF_4_∙∙∙HCHO	2.616(23.5%)	167.8	0.0092	0.0054	−6.64(−6.60)	−5.87	0.77
SeF_4_∙∙∙CH_3_CHO	2.533(25.9%)	169.4	0.0133	0.0078	−8.49(−7.80)	−7.22	1.27
SeF_4_∙∙∙CH_3_COCH_3_	2.522(26.3%)	173.2	0.0137	0.0076	−9.17(−8.23)	−7.62	1.55
SeF_4_∙∙∙HCOOH	2.551(25.4%)	167.9	0.0069	−0.0019	−11.16(−11.19)	−8.47	2.69

^a^ The parenthesized values are the percent differences between *R*(Se∙∙∙O) and the sum of the van der Waals radii of two chalcogen atoms. ^b^ The parenthesized values are computed at the CCSD(T)/CBS level.

**Table 3:** Topological parameters (a.u) at the Se∙∙∙O and H∙∙∙F BCPs, together with the energies (*E*_NCI_, kcal/mol) of these intermolecular interactions identified by the QTAIM analyses.

Heterodimer	BCP	*E*_NCI_	*ρ*	∇^2^*ρ*	*G*	*V*	*H*	|*G*/*V*|
SeF_2_∙∙∙H_2_O	Se∙∙∙O	−6.57 *^e^*	0.0306	0.0992	0.0252	−0.0255	−0.0004	0.9855
SeF_2_∙∙∙CH_3_OH	Se∙∙∙O	−9.19 *^e^*	0.0407	0.1167	0.0329	−0.0366	−0.0037	0.8982
SeF_2_∙∙∙CH_3_OCH_3_	Se∙∙∙O	−10.97 *^e^*	0.0472	0.1243	0.0376	−0.0442	−0.0066	0.8515
SeF_2_∙∙∙C_2_H_4_O	Se∙∙∙O	−9.27 *^e^*	0.0412	0.1175	0.0332	−0.0370	−0.0038	0.8970
SeF_2_∙∙∙HCHO	Se∙∙∙O	−7.22 *^e^*	0.0333	0.1051	0.0273	−0.0283	−0.0010	0.9644
	H∙∙∙F	−1.73 *^f^*	0.0111	0.0499	0.0105	−0.0085	0.0020	1.2350
SeF_2_∙∙∙CH_3_CHO	Se∙∙∙O	−8.11 *^e^*	0.0368	0.1115	0.0300	−0.0321	−0.0021	0.9346
	H∙∙∙F	−1.83 *^f^*	0.0115	0.0514	0.0108	−0.0088	0.0020	1.2312
SeF_2_∙∙∙CH_3_COCH_3_	Se∙∙∙O	−8.44 *^e^*	0.0383	0.1121	0.0307	−0.0334	−0.0027	0.9189
	H∙∙∙F	−1.16 *^f^*	0.0085	0.0377	0.0079	−0.0065	0.0015	1.2294
SeF_2_∙∙∙HCOOH	Se∙∙∙O	−7.35 *^e^*	0.0334	0.1078	0.0279	−0.0288	−0.0009	0.9678
	H∙∙∙F	−5.63 *^f^*	0.0286	0.1064	0.0271	−0.0276	−0.0005	0.9824
SeF_4_∙∙∙H_2_O	Se∙∙∙O	−5.24 *^e^*	0.0268	0.0837	0.0204	−0.0199	0.0005	1.0269
SeF_4_∙∙∙CH_3_OH	Se∙∙∙O	−8.52 *^e^*	0.0412	0.1058	0.0301	−0.0338	−0.0037	0.8912
	H∙∙∙F	−0.57 *^f^*	0.0059	0.0241	0.0050	−0.0041	0.0010	1.2413
SeF_4_∙∙∙CH_3_OCH_3_	Se∙∙∙O	−9.37 *^e^*	0.0450	0.1075	0.0321	−0.0374	−0.0053	0.8589
	H∙∙∙F_a1_ *^a^*	−1.21 *^f^*	0.0087	0.0345	0.0074	−0.0061	0.0013	1.2068
	H∙∙∙F_a2_ *^b^*	−1.21 *^f^*	0.0087	0.0347	0.0074	−0.0061	0.0013	1.2070
SeF_4_∙∙∙C_2_H_4_O	Se∙∙∙O	−9.47 *^e^*	0.0450	0.1107	0.0328	−0.0378	−0.0051	0.8655
SeF_4_∙∙∙HCHO	Se∙∙∙O	−5.84 *^e^*	0.0303	0.0870	0.0221	−0.0224	−0.0003	0.9848
	H∙∙∙F	−1.37 *^f^*	0.0095	0.0394	0.0084	−0.0069	0.0015	1.2116
SeF_4_∙∙∙CH_3_CHO	Se∙∙∙O	−7.19 *^e^*	0.0363	0.0970	0.0262	−0.0281	−0.0019	0.9309
	H∙∙∙F	−1.37 *^f^*	0.0094	0.0392	0.0084	−0.0069	0.0014	1.2054
SeF_4_∙∙∙CH_3_COCH_3_	Se∙∙∙O	−7.28 *^e^*	0.0359	0.1015	0.0270	−0.0286	−0.0016	0.9443
	H∙∙∙F_a_ *^c^*	−1.52 *^f^*	0.0101	0.0392	0.0083	−0.0069	0.0014	1.2098
	H∙∙∙F_e_ *^d^*	−0.87 *^f^*	0.0072	0.0311	0.0064	−0.0051	0.0013	1.2588
SeF_4_∙∙∙HCOOH	Se∙∙∙O	−6.79 *^e^*	0.0338	0.0975	0.0254	−0.0264	−0.0010	0.9608
	H∙∙∙F	−7.38 *^f^*	0.0364	0.1172	0.0335	−0.0376	−0.0042	0.8894

*^a^* F_a1_ indicates the top axial F atom of SeF_4_ in Figure S2. *^b^* F_a2_ indicates the bottom axial F atom of SeF_4_ in Figure S2. *^c^* F_a_ indicates the top axial F atom of SeF_4_ in Figure S2. *^d^* F_e_ indicates the left equatorial F atom of SeF_4_ in Figure S2. *^e^* Energy of each ChB was estimated using the formula: *E*_NCI_ = 0.375 × *V* − 0.5655. *^f^* Energy of each HB was estimated using the formula: *E*_NCI_ = −223.08 × *ρ* + 0.7423.

**Table 4:** *E*^(2)^ Values (kcal/mol) for the donor–acceptor orbital interactions involving the Se∙∙∙O ChBs in the examined heterodimers.

	LP(O) → σ*(Se–F^1^)	LP(O) → σ*(Se–F^2^)	LP(Se) → σ*(O–H/C)		LP(O) → σ*(Se–F^1^)	LP(O) → σ*(Se–F^2^)	LP(Se) → σ*(O–H/C)
SeF_2_				SeF_4_			
H_2_O	11.52	1.51	0.29/0.30	H_2_O	5.82	1.72	0.33
CH_3_OH	15.88	2.38	0.21/0.82	CH_3_OH	12.18	3.31	0.07/0.74
CH_3_OCH_3_	18.12	2.86	0.76/0.76	CH_3_OCH_3_	13.32	3.59	0.63/0.63
C_2_H_4_O	16.23	2.58	0.54/0.54	C_2_H_4_O	13.93	3.90	0.49/0.49
HCHO	13.14	1.99	0.86(0.86) ^a^	HCHO	7.79	2.17	(0.58)
CH_3_CHO	15.55	2.36	0.87(0.87)	CH_3_CHO	10.89	3.02	0.60
CH_3_COCH_3_	16.77	2.29	0.89(0.30)	CH_3_COCH_3_	11.12	2.96	0.95
HCOOH	13.93	2.50	0.88(0.94)	HCOOH	10.12	2.72	0.23(0.58)

^a^ The values in parentheses are related to the LP(Se) → π*(O=C) orbital interactions.

## Data Availability

The data presented in this study are available in the article and its Supplementary Materials.

## Supplementary Materials

The following supporting information can be downloaded at: https://www.mdpi.com/article/10.3390/molecules29235739/s1, Figure S1: The MEP maps of eight oxygen-bearing Lewis bases on the 0.001 electron/Bohr^3^ isosurface. The negative electrostatic potentials are indicated by the blue areas and the positive electrostatic potentials are represented by the red areas; Figure S2: QTAIM analysis maps of the targeted heterodimers. The orange and yellow dots indicate the bond critical points and ring critical points, respectively. The brown lines denote the bond paths; Figure S3: NBO plots of the donor–acceptor interactions for the sixteen studied heterodimers; Figure S4: Correlation plots between interaction energies (*E*_int_) and the total interaction energies (*E*_total_) of the studied heterodimers obtained by the SAPT analysis; Table S1: Cartesian coordinates of the heterodimers of SeF_2_ and SeF_4_ with O-containing Lewis bases in their principal inertial axis systems at the MP2/aug-cc-pVTZ level of theory; Table S2: SAPT analysis findings (kcal/mol) on the attractive and repulsive components for the SeF_2_ and SeF_4_ heterodimers.
