# Supplementary material for: Unraveling the Strength and Nature of Se∙∙∙O Chalcogen Bonds: A Comparative Study of SeF2 and SeF4 Interactions with Oxygen-Bearing Lewis Bases
Source: Molecules. 2024 Dec 5;29(23):5739. doi: 10.3390/molecules29235739 (PMC11643493; doi:10.3390/molecules29235739)
Supplement: Supplementary file 1 [file molecules-29-05739-s001.zip › molecules-3330137-supplementary.pdf]

Supporting Materials

Unraveling the Strength and Nature of Se···O Chalcogen Bonds: A Comparative Study of SeF2 and SeF4 Interactions with Oxygen-Bearing Lewis Bases

Renhua Chen [1,†], Fengying Lei [1,†], Deze Jin [1], Ke Peng [1], Qingyu Liu [1], Yeshuang Zhong [1], Liang Hong [1], Xiaolong Li [2], Zhu Zeng [1,*] and Tao Lu [1,*]

[1] School of Basic Medical Sciences/School of Biology and Engineering, Guizhou Medical University, Guiyang, 550025, China

[2] Department of Chemistry, Shanghai Key Laboratory of Molecular Catalysis and Innovative Materials, Fudan University, Shanghai 200433, China

† These authors contributed equally to this work.

Corresponding Authors

* Zhu Zeng: zengzhu@gmc.edu.cn;   * Tao Lu: lutao0409@gmc.edu.cn;

Contents:

Supplementary figures

Figure S1. The MEP maps of eight oxygen-bearing Lewis bases on the 0.001 electron/Bohr^3 isosurface. The negative electrostatic potentials are indicated by the blue areas and the positive electrostatic potentials are represented by the red areas.

Figure S2. QTAIM analysis maps of the targeted heterodimers. The orange and yellow dots indicate the bond critical points and ring critical points, respectively. The brown lines denote the bond paths.

Figure S3. NBO plots of the donor-acceptor interactions for the sixteen studied heterodimers.

Figure S4. Correlation plots between interaction energies (E_{int}) and the total interaction energies (E_{total}) of the studied heterodimers obtained by the SAPT analysis.

Supplementary tables

Table S1. Cartesian coordinates of the heterodimers of SeF_2 and SeF_4 with O-containing Lewis bases in their principal inertial axis systems at the MP2/aug-cc-pVTZ level of theory.

Table S2. SAPT analysis findings (kcal/mol) on the attractive and repulsive components for the SeF_2 and SeF_4 heterodimers.

Supplementary figures

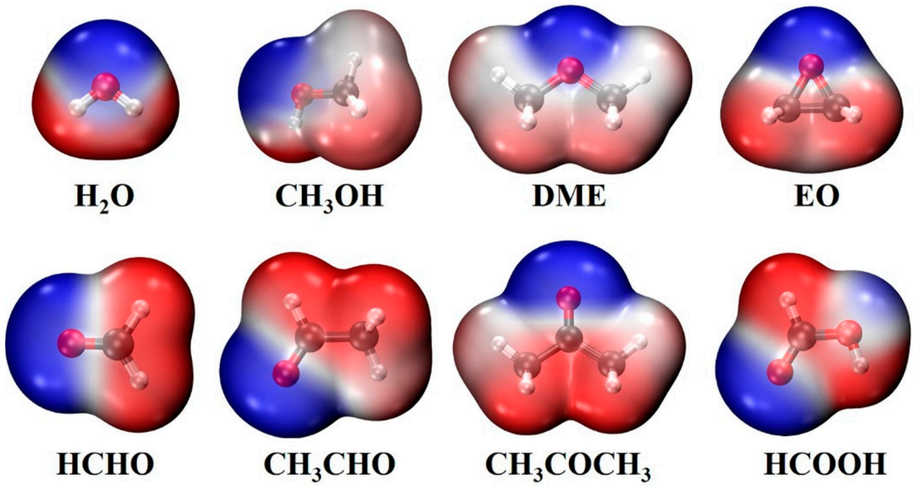

Figure S1. The MEP maps of eight oxygen-bearing Lewis bases on the 0.001 electron/Bohr^3 isosurface. The negative electrostatic potentials are indicated by the blue areas and the positive electrostatic potentials are represented by the red areas.

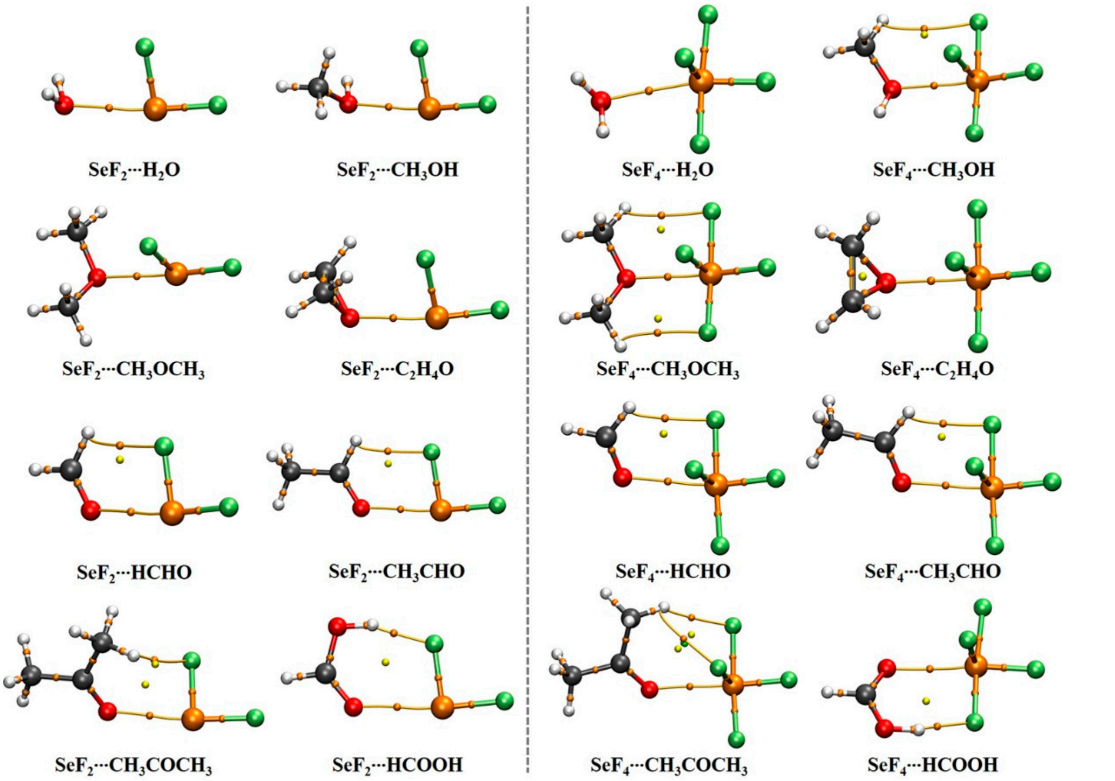

Figure S2. QTAIM analysis maps of the targeted heterodimers. The orange and yellow dots indicate the bond critical points and ring critical points, respectively. The brown lines denote the bond paths.

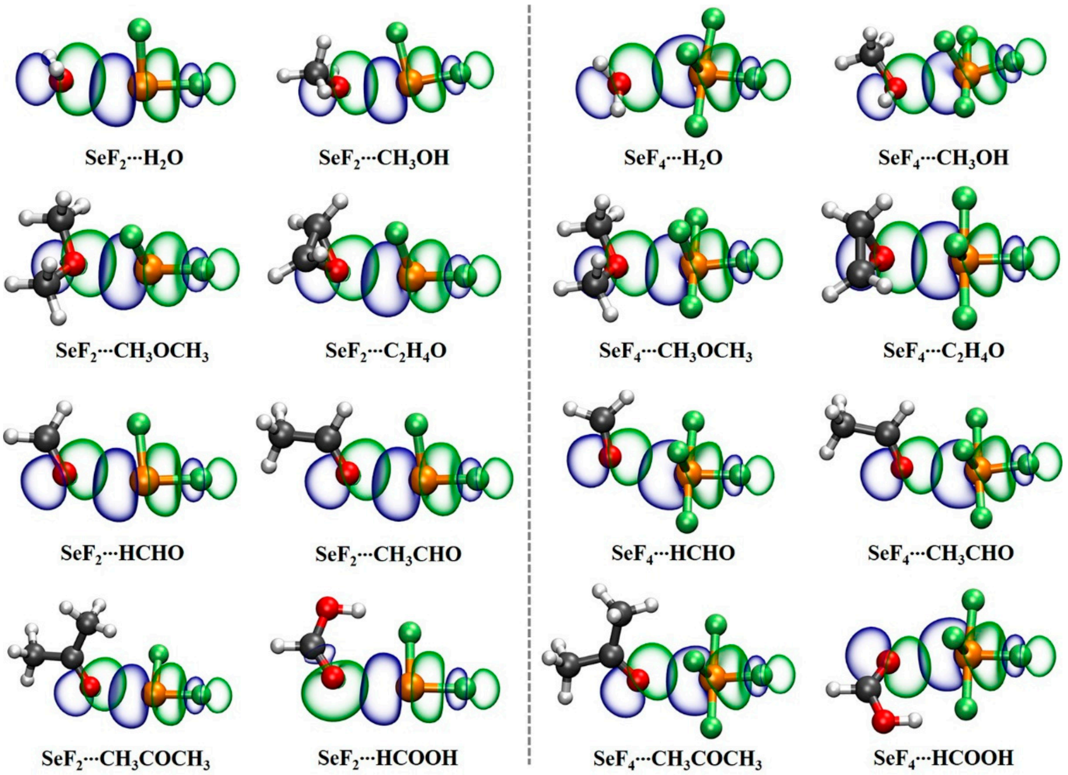

Figure S3. NBO plots of the donor-acceptor interactions for the sixteen studied heterodimers.

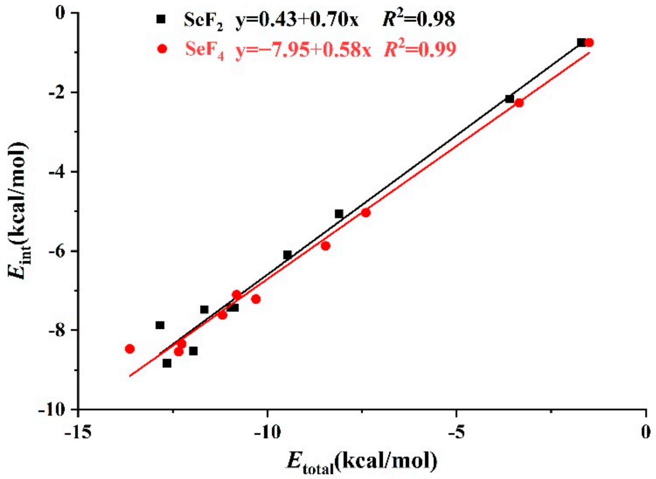

Figure S4. Correlation plots between interaction energies (E_{int}) and the total interaction energies (E_{total}) of the studied heterodimers obtained by the SAPT analysis.

Supplementary tables

Table S1. Cartesian coordinates of the heterodimers of SeF_2 and SeF_4 with O-containing Lewis bases in their principal inertial axis systems at the MP2/aug-cc-pVTZ level of theory.

$SeF_2 \cdots H_2O$			
Atom	a (Å)	b (Å)	c (Å)
O	2.336361	-0.313968	0.000434
H	2.648021	0.186435	0.762972
H	2.650959	0.183725	-0.762663
Se	-0.213305	-0.290209	-0.000127
F	-1.949975	-0.092532	0.000223
F	0.090254	1.426831	-0.000165

$SeF_4 \cdots H_2O$			
Atom	*a* (Å)	*b* (Å)	*c* (Å)
O	2.431748	-0.430107	-0.318231
H	2.853756	-1.041905	0.294669
H	2.704858	0.446407	-0.020910
Se	-0.192067	-0.004140	-0.251247
F	0.466350	1.646706	-0.003818
F	0.175565	-0.246684	1.380555
F	-1.727205	0.669337	-0.010868
F	-0.968303	-1.605235	-0.164261

$SeF_2 \cdots CH_3OH$			
Atom	*a* (Å)	*b* (Å)	*c* (Å)
C	-2.607569	0.190452	0.514026
H	-2.369925	-0.224976	1.487771
H	-3.668393	0.057130	0.310544
H	-2.347760	1.247084	0.498735
O	-1.818129	-0.545086	-0.435128
H	-1.987419	-0.187935	-1.315001
Se	0.561014	-0.312541	-0.017197
F	2.247501	0.075268	0.277548
F	0.140216	1.363960	-0.277601

$SeF_4 \cdots CH_3OH$			
Atom	*a* (Å)	*b* (Å)	*c* (Å)
C	-2.825927	0.231101	0.184438
H	-2.597630	1.248670	-0.114680
H	-2.700158	0.137373	1.260956
H	-3.845616	-0.017497	-0.101914
O	-1.895006	-0.611632	-0.518351
H	-1.989506	-1.516383	-0.195181
Se	0.485497	0.003463	-0.275224
F	0.073526	1.747217	-0.209318
F	2.086682	0.390867	0.149506
F	-0.049256	-0.016522	1.335065
F	0.860341	-1.728614	0.007927

$SeF_2 \cdots CH_3OCH_3$			
Atom	*a* (Å)	*b* (Å)	*c* (Å)
O	-1.576027	0.000713	-0.504978
C	-2.154246	1.178223	0.059043
H	-1.660712	2.025871	-0.405822
H	-3.223466	1.202738	-0.156960
H	-1.991610	1.199999	1.137849
C	-2.154219	-1.178311	0.055897
H	-3.223437	-1.202280	-0.160179
H	-1.660658	-2.024704	-0.411219
H	-1.991593	-1.202955	1.134643
Se	0.780520	0.000501	-0.315494
F	2.501676	-0.000012	0.056741
F	0.350859	-0.002309	1.380891

$SeF_4 \cdots CH_3OCH_3$			
Atom	*a* (Å)	*b* (Å)	*c* (Å)
O	-1.754671	0.003153	-0.472843
C	-2.365658	1.177717	0.065469
H	-1.834173	2.031680	-0.341228
H	-3.415247	1.208882	-0.229952
H	-2.286212	1.179904	1.154035
C	-2.367060	-1.178464	0.048042
H	-3.418079	-1.201392	-0.243055
H	-1.840325	-2.027190	-0.375440
H	-2.282669	-1.199582	1.136053
Se	0.673710	-0.001538	-0.277241
F	0.670157	1.786189	-0.112099
F	2.329229	-0.001080	0.128549
F	0.178880	0.006879	1.347350
F	0.666649	-1.787629	-0.094085

$SeF_2 \cdots C_2H_4O$			
Atom	*a* (Å)	*b* (Å)	*c* (Å)
C	2.420997	0.184317	-0.731195
C	2.420975	0.184474	0.731214
O	1.575721	-0.737790	0.000094
H	1.875308	0.959788	-1.249456
H	3.226191	-0.300643	-1.264053
H	1.875273	0.960057	1.249292
H	3.226152	-0.300376	1.264199
Se	-0.805535	-0.327870	0.000018
F	-0.231338	1.326228	-0.000135
F	-2.487809	0.175808	-0.000029

$SeF_4 \cdots C_2H_4O$			
Atom	*a* (Å)	*b* (Å)	*c* (Å)
C	2.582724	0.730922	0.188036
C	2.583545	-0.730864	0.189363
O	1.715338	-0.001332	-0.718744
H	2.051138	1.257655	0.967362
H	3.374059	1.263641	-0.318801
H	2.052633	-1.256679	0.969756
H	3.375518	-1.263636	-0.316422
Se	-0.673558	0.000050	-0.289461
F	-0.647747	-1.781796	-0.102750
F	-2.290844	0.001511	0.246681
F	-0.047379	-0.000682	1.294454
F	-0.644330	1.781812	-0.102234

$SeF_2 \cdots HCHO$			
Atom	*a* (Å)	*b* (Å)	*c* (Å)
C	2.730423	0.252423	-0.000097
O	1.945846	-0.680911	-0.000181
H	3.810647	0.068313	-0.000088
H	2.377363	1.288802	0.000178
Se	-0.535537	-0.316079	0.000129
F	0.011105	1.342983	0.000061
F	-2.225443	0.137276	-0.000333

$SeF_4 \cdots HCHO$			
Atom	*a* (Å)	*b* (Å)	*c* (Å)
C	-2.849899	0.051367	0.111598
O	-2.079035	-0.720002	-0.433921
H	-3.931135	-0.124638	0.076714
H	-2.485135	0.936772	0.644500
Se	0.433621	-0.012553	-0.265804
F	1.206867	-1.596658	-0.038024
F	-0.057870	-0.173062	1.345594
F	1.938388	0.692695	0.073383
F	-0.264626	1.639967	-0.145630

$SeF_2 \cdots CH_3CHO$			
Atom	*a* (Å)	*b* (Å)	*c* (Å)
C	2.257976	0.232600	0.000196
O	1.449477	-0.685273	-0.000480
H	1.894103	1.269092	0.000682
Se	-0.986164	-0.314944	-0.000047
F	-0.452788	1.349529	-0.000413
F	-2.680659	0.138727	0.000505
C	3.732618	0.023343	0.000292
H	3.975591	-1.034546	-0.000290
H	4.160899	0.513389	-0.875374
H	4.160613	0.512356	0.876675

$SeF_4 \cdots CH_3CHO$			
Atom	*a* (Å)	*b* (Å)	*c* (Å)
C	-2.419599	0.110235	0.089537
O	-1.641466	-0.653294	-0.466927
H	-2.023535	0.962537	0.659924
Se	0.801946	-0.013892	-0.269124
F	1.454129	-1.650614	-0.014120
F	0.297436	-0.119894	1.345325
F	2.344737	0.597517	0.100667
F	0.191832	1.677970	-0.170886
C	-3.898643	-0.046471	0.036882
H	-4.336073	0.860618	-0.382965
H	-4.178363	-0.913165	-0.553628
H	-4.280217	-0.138706	1.054921

$SeF_2 \cdots CH_3COCH_3$			
Atom	*a* (Å)	*b* (Å)	*c* (Å)
C	2.113858	-0.083312	-0.072994
O	1.198905	-0.879433	0.124287
Se	-1.175619	-0.333698	-0.120814

$SeF_4 \cdots CH_3COCH_3$			
Atom	*a* (Å)	*b* (Å)	*c* (Å)
C	-2.426704	-0.097018	-0.075486
O	-1.417406	-0.700075	-0.431524
Se	1.030541	-0.113375	-0.271196

F	-0.692578	0.962960	0.943864
F	-2.844593	0.215008	-0.157933
C	3.535207	-0.477636	0.200744
H	3.574114	-1.448215	0.684905
H	4.080605	-0.511285	-0.743859
H	4.016646	0.278897	0.820978
C	1.869643	1.308225	-0.576750
H	1.827240	1.976539	0.285538
H	2.685943	1.636469	-1.217218
H	0.917546	1.363405	-1.096338

F	1.411111	-1.711499	0.415688
F	0.480688	0.265150	1.286342
F	2.644504	0.329629	0.030283
F	0.710240	1.624075	-0.627164
C	-2.368661	1.261094	0.559350
H	-2.412510	1.124122	1.642251
H	-1.442882	1.768182	0.308713
H	-3.231505	1.857657	0.269038
C	-3.781808	-0.716561	-0.251196
H	-4.344219	-0.651397	0.680431
H	-4.334124	-0.144866	-0.998992
H	-3.689741	-1.749617	-0.570933

$SeF_2\cdots HCOOH$			
Atom	*a* (Å)	*b* (Å)	*c* (Å)
Se	0.875904	-0.334260	-0.000261
F	2.483586	0.344687	0.000181
F	0.131689	1.269162	-0.000202
C	-2.535096	-0.402510	0.000541
O	-1.512234	-1.064140	0.000421
O	-2.599745	0.919862	0.000223
H	-3.530286	-0.848838	0.000885
H	-1.681518	1.258315	-0.000218

$SeF_4\cdots HCOOH$			
Atom	*a* (Å)	*b* (Å)	*c* (Å)
Se	-0.689575	-0.027933	-0.258778
F	0.292017	1.540077	-0.185835
F	-0.271298	-0.163423	1.367887
F	-2.051088	0.944968	0.003429
F	-1.702142	-1.445602	0.002105
C	2.665956	-0.581928	-0.087416
O	1.606339	-1.138467	-0.330448
O	2.828228	0.704610	0.154764
H	3.614893	-1.118830	-0.047180
H	1.950976	1.146799	0.087314

Table S2. SAPT analysis findings (kcal/mol) on the attractive and repulsive components for the SeF_2 and SeF_4 heterodimers

complexes	E_{elec}	E_{ind}	E_{disp}	E_{ex-re}	E_{total}
SeF_4···H_2O	−17.42（56%）[a]	−7.35（24%）	−6.30（20%）	22.97	−8.11
SeF_2···CH_3OH	−25.03（54%）	−11.99（25%）	−9.65（21%）	35.80	−10.86
SeF_2···DME	−29.84（52%）	−15.34（27%）	−12.65（21%）	44.98	−12.65
SeF_2···EO	−24.48（51%）	−12.92（27%）	−10.93（22%）	36.38	−11.95
SeF_2···HCHO	−18.22（51%）	−9.37（27%）	−7.98（22%）	26.10	−9.47
SeF_2···CH_3CHO	−21.35（51%）	−11.32（27%）	−8.88（22%）	30.58	−10.97
SeF_2···CH_3COCH_3	−24.11（51%）	−12.62（26%）	−10.79（23%）	35.85	−11.67
SeF_2···HCOOH	−24.43（51%）	−12.99（27%）	−10.16（22%）	34.74	−12.84
SeF_4···H_2O	−16.54（58%）	−6.11（21%）	−6.13（21%）	20.14	−8.63
SeF_4···CH_3OH	−26.24（53%）	−12.71（26%）	−10.20（21%）	36.54	−12.61
SeF_4···DME	−29.09（51%）	−15.24（26%）	−13.09（23%）	43.01	−14.40
SeF_4···EO	−27.73（50%）	−15.29（28%）	−12.21（22%）	40.93	−14.31
SeF_4···HCHO	−17.27（51%）	−8.24（25%）	−8.00（24%）	23.64	−9.87
SeF_4···CH_3CHO	−21.96（51%）	−11.30（27%）	−9.47（22%）	30.72	−12.01
SeF_4··CH_3COCH_3	−23.58（52%）	−11.64（28%）	−10.38（23%）	32.55	−13.05
SeF_4···HCOOH	−29.48（52%）	−15.27（27%）	−11.64（21%）	40.49	−15.90

[a] The values in parentheses indicate the contribution of each attractive component to the total attractive interaction energy.
